# The Usefulness of the BD MAX MDR-TB Molecular Test in the Rapid Diagnosis of Multidrug-Resistant Tuberculosis

**DOI:** 10.3390/pathogens14060602

**Published:** 2025-06-19

**Authors:** Tomasz Bogiel, Edyta Dolska, Małgorzata Zimna, Kornelia Nakonowska, Dorota Krawiecka, Renata Żebracka, Maciej Pochowski, Agnieszka Krawczyk

**Affiliations:** 1 Department of Propaedeutics of Medicine and Infection Prevention Ludwik Rydygier Collegium Medicum in Bydgoszcz, Nicolaus Copernicus University in Toruń, 9 Maria Skłodowska-Curie Street, 85-094 Bydgoszcz, Poland; 2 Clinical Microbiology Laboratory, Dr. Antoni Jurasz University Hospital No. 1 in Bydgoszcz, 9 Maria Skłodowska-Curie Street, 85-094 Bydgoszcz, Poland; 3 Department of Microbiological Diagnostics, Kujawsko-Pomorskie Pulmonology Centre in Bydgoszcz, 85-326 Bydgoszcz, Poland; e.dolska@kpcp.pl (E.D.); m.zimna@kpcp.pl (M.Z.); k.nakonowska@kpcp.pl (K.N.); dorota.krawiecka@kpcp.pl (D.K.); r.zebracka@kpcp.pl (R.Ż.); m.pochowski@kpcp.pl (M.P.); 4 Department of Molecular Medical Microbiology, Chair of Microbiology, Jagiellonian University Medical College, 31-121 Kraków, Poland

**Keywords:** BD MAX MDR-TB, isoniazid, multidrug-resistant tuberculosis, *Mycobacterium tuberculosis* complex, rifampicin, tuberculosis

## Abstract

Tuberculosis (TB), primarily caused by *Mycobacterium tuberculosis* complex (MTBC), remains a global health challenge and can lead to severe pulmonary and extrapulmonary complications. Multidrug-resistant TB (MDR-TB) poses additional challenges, requiring advanced diagnostic and treatment strategies. This study evaluates the BD MAX MDR-TB molecular test for a rapid diagnosis of MDR-TB, detecting resistance to rifampicin (RIF) and isoniazid (INH). The BD MAX MDR-TB test, utilizing real-time PCR, was used to analyze specimens collected from TB-suspected patients, identifying MTB DNA and mutations associated with rifampicin and isoniazid resistance. Results were compared with traditional drug susceptibility testing, and 79 out of 638 samples tested were positive for MTB DNA, with 65 showing a sufficient amount of genetic material for resistance gene identification. The BD MAX test showed a 100% correlation with phenotypic rifampicin resistance, though discrepancies were noted for isoniazid resistance, with a 93% concordance. The BD MAX MDR-TB test is an effective tool for a rapid diagnosis of MDR-TB, especially for rifampicin resistance. However, it may not detect certain mutations related to isoniazid resistance. Complementary tests like Xpert MTB/XDR or whole-genome sequencing could improve diagnostic accuracy and support more effective TB control strategies.

## 1. Introduction

Tuberculosis (TB) is a bacterial infection primarily caused by *Mycobacterium tuberculosis*. It mainly targets the lungs but can also spread to other organs, leading to severe health complications. The disease is transmitted through airborne droplets, and common symptoms include persistent cough, chest pain, fatigue, fever, night sweats, and weight loss [1] Tuberculosis remains a significant global health issue, with *Mycobacterium tuberculosis* complex (MTBC) presenting significant challenges in both its diagnosis and therapeutic management [2]. This complex belongs to the *Mycobacteriaceae* family, which includes a diverse group of bacteria exhibiting various levels of pathogenicity in humans and animals. These bacteria are generally non-spore-forming, aerobic, and non-motile, characterized by a unique acid-fast cell wall rich in mycolic acids, which makes them resistant to many conventional treatments [3]. The *Mycobacterium* genus comprises several closely related species, with *M. tuberculosis* being the most significant pathogen.

According to the latest World Health Organization (WHO) Global Tuberculosis Report 2024 [4], 10.8 million people were diagnosed with tuberculosis globally in 2023, reflecting a slight increase from 10.7 million in 2022. This corresponds to an incidence rate of 134 cases per 100,000 people. Among these cases, approximately 6.1% involved people with HIV. The highest percentage of TB cases was recorded in the South-East Asia Region (45%), Africa (24%), and the Western Pacific (17%). Lower proportions were observed in the Eastern Mediterranean (8.6%), the Americas (3.2%), and Europe (2.1%). Despite some progress, including a decline in TB deaths from 1.32 million in 2022 to 1.25 million in 2023, tuberculosis has once again become the leading cause of death from a single infectious agent, surpassing COVID-19. According to epidemiological data from Poland [5], in 2023, 4231 cases of tuberculosis were reported, including 4077 cases of pulmonary TB. The overall incidence rate was 11.2 per 100,000 population, with a higher prevalence among men (17.2 per 100,000) than women (5.6 per 100,000). Additionally, 45 cases were recorded among children and 60 among adolescents.

Multidrug-resistant (MDR) strains of *M. tuberculosis* have emerged as a serious concern, necessitating advanced diagnostic techniques and effective treatment strategies. Multidrug-resistant tuberculosis continues to pose a challenge, with 89 MDR-TB cases identified in 2023 in Poland, alongside 11 cases resistant to rifampicin [5]. Although these numbers indicate a slight decline from 2022, when 98 MDR-TB cases were recorded, the implications for public health are profound, as MDR-TB is associated with prolonged treatment, higher costs, and increased mortality [2]. This highlights the need for effective diagnostic and treatment strategies.

Standard tuberculosis treatment regimens consist of four primary first-line medications: isoniazid (INH), rifampicin (RIF), pyrazinamide, and ethambutol. While streptomycin was once considered a first-line medication for tuberculosis, it is no longer commonly used due to increasing drug resistance and side effects [6,7]. The choice of medications and the duration of tuberculosis treatment depend on form of the disease (active or latent), drug susceptibility, the patient’s immune status (e.g., HIV co-infection), age, body weight, and potential drug interactions. A major concern in TB therapy is the development of multidrug-resistant tuberculosis (MDR-TB), which is characterized by resistance to at least isoniazid and rifampicin [8].

Isoniazid is a prodrug activated by bacterial catalase-peroxidase (katG), resulting in the formation of an inactive analogue of nicotinamide adenine dinucleotide (NAD). This leads to the inhibition of mycolic acid synthesis, a crucial element of the mycobacterial cell wall, resulting in structural damage and ultimately the death of the bacterial cell. Mutations in the *katG* gene are the primary mechanism for INH resistance. Additionally, alterations in genes such as *inhA*, *ahpC*, *kasA*, *ndh*, *iniABC*, *fadE*, *furA*, *Rv1592c*, and *Rv1772* also contribute to this resistance pattern. Recent studies have also highlighted the involvement of efflux genes in isoniazid resistance, as well as substitutions in *nat*, *fabD*, and *accD* genes [9].

Rifampicin is a semi-synthetic antibiotic that works by binding to DNA-dependent RNA polymerase, temporarily blocking its activity, which in turn disrupts bacterial protein synthesis and transcription processes. Rifampicin exhibits strong bactericidal activity against *M. tuberculosis*, found both intracellularly and extracellularly, as well as against atypical mycobacteria and *M. leprae* [6]. Resistance to rifampicin in tuberculosis is primarily caused by mutations in the *rpoB* gene, which encodes the β subunit of RNA polymerase in *M. tuberculosis*. These mutations most frequently occur in the rifampicin resistance determining region (RRDR) and result in structural changes in the enzyme, preventing rifampicin from effectively binding to RNA polymerase. As a consequence, the bacteria become resistant to the antibiotic. Approximately 90–95% of RIF-resistant isolates have been identified as having mutations in the *rpoB* gene. However, the resistance mechanism in the remaining 5% is unclear, suggesting that other factors, such as lowered cell wall permeability or an enhanced efflux pump, may play a role [10].

**Figure 1:**
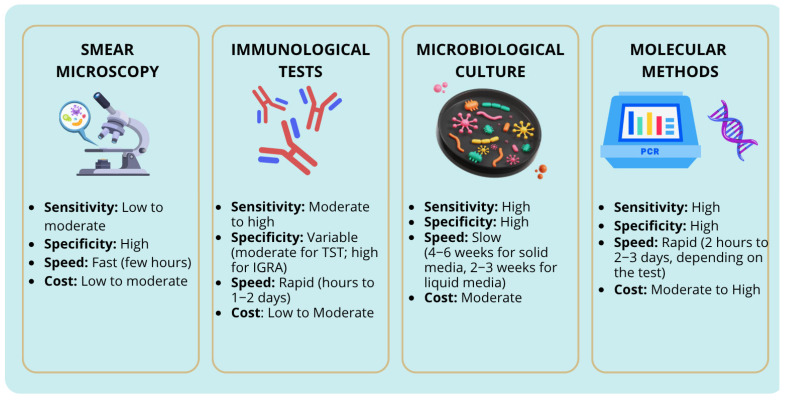
Comparison of diagnostic methods for tuberculosis. The figure illustrates the comparison of various diagnostic techniques used in the detection of tuberculosis, including culture, smear microscopy, molecular methods, and immunological tests. Each method is assessed based on sensitivity, specificity, speed and cost; IGRA—interferon-gamma release assay; TST—tuberculin skin test.

Traditional methods for diagnosing tuberculosis include smear microscopy, culture, immunological tests, and nucleic acid amplification techniques (NAAT) (Figure 1). Although culture remains the gold standard due to its high sensitivity, it is time-consuming, requiring up to 6 weeks for results. In contrast, molecular diagnostic techniques such as PCR allow for the faster detection of *M. tuberculosis* DNA directly from clinical specimens, providing results within a few hours and significantly improving early diagnosis and patient management. However, positive molecular results still require confirmation by culture and susceptibility testing, especially in cases with discrepant findings.

Among molecular diagnostic platforms, the BD MAX MDR-TB assay offers a practical and efficient solution for the rapid detection of multidrug-resistant tuberculosis. It is a relatively new and WHO-approved molecular test for the rapid diagnosis of multidrug-resistant tuberculosis by detecting MTB DNA along with simultaneous resistance to antibiotics. In comparison to the GeneXpert MTB/RIF system, which primarily detects rifampicin resistance, BD MAX identifies resistance to both rifampicin and isoniazid [11]. Although there is a newer version of the GeneXpert test, i.e., the MTB/XDR assay, which extends detection to isoniazid and selected second-line drugs, its availability remains limited in many clinical settings. The MTB/XDR assay, introduced in 2021, requires upgraded GeneXpert platforms (10-color modules), making its adoption slower due to higher costs and infrastructure demands [12,13]. In turn, BD MAX features a high level of automation, allowing for the processing of up to 24 samples per run. The BD MAX MDR-TB assay is particularly well-suited for central laboratories where large numbers of samples are tested and there is a need for minimal operator hands-on time. The cost-effectiveness of the BD MAX system and its compatibility with various laboratory settings make it an attractive option for expanding tuberculosis diagnostics in low- and middle-income countries, where the burden of multidrug-resistant tuberculosis is particularly high [11]. While whole-genome sequencing (WGS) provides comprehensive resistance profiling, it remains technically challenging, cost-intensive, and less feasible for routine clinical use due to prolonged result delivery times (several days) and the need for specialized bioinformatics analysis [14]. Moreover, in low- and middle-income countries, the high costs, technical requirements, and need for specialized infrastructure make WGS even more difficult to implement, limiting its practicality in these regions. Therefore, BD MAX offers a valuable balance between diagnostic speed, range, automation, and cost-effectiveness 

A crucial aspect of tuberculosis control is not only the rapid detection of the disease but also the effective identification of MDR strains. Traditional culture-based methods, while reliable, are time-consuming, requiring weeks to yield results. In contrast, molecular diagnostic techniques have demonstrated significant advantages in detecting drug-resistant MTB strains swiftly and accurately [15,16]. Implementing these technologies on a broader scale could substantially improve TB control efforts in Poland and worldwide.

In light of these challenges, effective control of multidrug-resistant tuberculosis requires continuous monitoring, improvements in diagnostic techniques, and the strengthening of public health measures. Solving this problem requires a coordinated effort from healthcare professionals, researchers and society to ensure early diagnosis, appropriate treatment and effective prevention strategies. Therefore, the aim of our study was to evaluate the usefulness of the BD MAX MDR-TB (Becton Dickinson, Franklin Lakes, NJ, USA) for the rapid detection of multidrug-resistant tuberculosis.

## 2. Materials and Methods

### 2.1. Patients

The research was carried out on 638 samples (for details of their origin see Supplementary Materials, Table S1) collected between January 2021 and September 2022 from patients suspected of tuberculosis, hospitalized in Kuyavian-Pomeranian Pulmonology Center in Bydgoszcz, Poland. All samples from the specified period, obtained from different patients, were included in the study if complete diagnostic data were available and the remaining volume of the clinical material after routine diagnostic procedures was sufficient to perform additional testing using the BD MAX system (Becton Dickinson, Franklin Lakes, NJ, USA). Inclusion criteria comprised suspected tuberculosis, availability of full diagnostic data, and sufficient sample volume for molecular analysis.

### 2.2. BD MAX MDR-TB Test

The BD MAX MDR-TB test, designed for use with the BD MAX System (Becton Dickinson, Franklin Lakes, NJ, USA), is an automated in vitro diagnostic assay intended for the qualitative detection of *M. tuberculosis* complex DNA in raw sputum samples or concentrated sputum sediments derived from induced or expectorated sputum specimens. In samples where MTBC DNA is detected, the BD MAX MDR-TB test can also identify mutations in the *rpoB* gene, associated with rifampicin resistance, as well as mutations in the *katG* gene and the *inhA* promoter region, which are linked to isoniazid resistance. This test uses qPCR to amplify specific target DNA sequences, along with fluorogenic hybridization probes to detect MTBC DNA and genetic mutations related to multidrug-resistant tuberculosis.

The results of 638 tests performed using the BD MAX MDR-TB (qPCR) test in the BD MAX system, in accordance with the manufacturer’s instructions, were analyzed. The methodology, briefly, was as follows: the sample collection tube (BD MAX MDR-TB) was labeled with a barcode sticker. The raw sputum sample was allowed to reach room temperature. The BD MAX STR reagent tube was opened, and the required volume of liquid was added to achieve a STR solution/sample ratio of 2:1. The container was closed, and the mixture was shaken vigorously 10 times. The sample mixture was incubated at room temperature for 5 min, followed by an additional vigorous shaking for 10 cycles. The sample treated with BD MAX STR reagent was incubated at room temperature for 25 min. Then, 2.5 mL of the STR-treated sputum sample was transferred into the labeled BD MAX MDR-TB sample tube using the provided transfer pipette. The BD MAX System was turned on, and the reagent strip was placed into the system rack. The sample tubes were placed into the corresponding rack positions, ensuring the barcode faced outward for easy scanning. The required number of BD PCR cartridges was loaded into the BD MAX System. The system lid was closed, and the reaction was started.

### 2.3. The Traditional Microscopy and Culture-Based Diagnosis

Samples were simultaneously cultured on liquid media using the BD BACTEC 960 MGIT system (Becton Dickinson, Franklin Lakes, NJ, USA) and on solid Löwenstein–Jensen media (Graso, Gdańsk, Poland). For the isolated strains, drug susceptibility was determined (for first-line drugs) on liquid media in the BD BACTEC 960 MGIT system using the SIRE (streptomycin, isoniazid, rifampicin and ethambutol) and pyrazinamide kits (Becton Dickinson Franklin Lakes, NJ, USA). The maximum duration of the SIRE test was 13 days, while the pyrazinamide test took up to 21 days. The correlation between the drug resistance results of *M. tuberculosis* obtained through molecular methods and AST was assessed.

## 3. Results

The results obtained using the BD MAX MDR-TB test were interpreted according to the manufacturer’s recommendations (BD MAX MDR-TB assay IFU, Becton Dickinson, Franklin Lakes, NJ, USA) and are summarized below (Table 1).

DNA of MTB was detected in 79 samples using the BD MAX system. Mutations were detected in 22 (27.8%) samples (Table 2), with confirmation in culture obtained for 71 of these samples.

In 65 of these samples, the amount of genetic material was sufficient for the identification of resistance genes (Table 3).

A complete correlation between the molecular results and AST results was obtained for rifampicin (Table 4). For all 15 samples with an *rpoB* mutation, the AST performed after culturing *M. tuberculosis* showed phenotypic resistance to rifampicin. In 14 of these samples, the BD MAX MDR-TB test also detected genotypic resistance to isoniazid. Additionally, no strains resistant to rifampicin were cultured from any of the samples without an *rpoB* mutation.

Out of 21 samples with a detected isoniazid resistance gene, phenotypic resistance to this drug was observed in the cultured strains. Similarly, from 32 samples without the detected isoniazid resistance gene, phenotypically sensitive strains were cultured. In the case of four samples, the BD MAX MDR-TB test did not detect isoniazid resistance genes; however, the AST results identified the cultured strains as phenotypically resistant (with 93% agreement for isoniazid) (Table 5).

To resolve these discrepancies, the Xpert MTB/XDR molecular test, which detected four isoniazid-related gene mutations, was used. This confirmed the absence of mutations in *inhA* and *katG*, while mutations in *ahpC* were detected in two strains, and mutations in *fabG1* were found in another two strains. The lack of correlation between the BD MAX MDR-TB test and AST regarding isoniazid resistance in these four strains is therefore attributed to the presence of other gene mutations associated with isoniazid resistance, which are not detected by the BD MAX MDR-TB test.

Additionally, three of these samples were both genotypically and phenotypically sensitive to rifampicin (not MDR strains), while one strain, which showed an *rpoB* mutation through the BD MAX MDR-TB test, was also phenotypically resistant to rifampicin (MDR strain).

## 4. Discussion

The obtained results confirm the high effectiveness of the BD MAX system in detecting MTB DNA and identifying resistance genes for rifampicin and isoniazid. The complete correlation between molecular and phenotypic results for rifampicin suggests that the BD MAX MDR-TB test is a reliable tool for identifying *rpoB* mutations, which are crucial for resistance to this drug. All samples in which an *rpoB* mutation was detected were phenotypically resistant to rifampicin, and in samples without this mutation, phenotypic resistance was not observed. This high concordance (100%) indicates the excellent predictive value of the BD MAX test in detecting rifampicin resistance, which is crucial since rifampicin is a key component of the first-line therapy of tuberculosis-suffering patients. However, the observed discrepancies in the case of isoniazid indicate slight limitations in detecting all potential mutations associated with resistance to this antibiotic. For isoniazid, a 93% concordance was obtained between the BD MAX test results and AST. Four samples in which BD MAX MDR-TB did not detect resistance were phenotypically resistant to isoniazid. Further analysis using the Xpert MTB/XDR test revealed the presence of mutations in the *ahpC* and *fabG1* genes, which are associated with isoniazid resistance but are not included in the detection range of the BD MAX MDR-TB test. These findings underscore the clinical importance of understanding the mutation spectrum in *M. tuberculosis* and adapting diagnostic algorithms accordingly. In settings with a high burden of drug-resistant TB, integrating BD MAX with additional molecular or sequencing-based methods can optimize detection and guide more effective treatment strategies.

Our results are consistent with those obtained by other researchers. Ciesielczuk et al. [17] analyzed 128 samples using BD MAX MDR-TB and compared them with smear and liquid culture results, as well as with the Xpert MTB/RIF assay. In their study, the BD MAX MDR-TB test demonstrated a concordance of 92% with the reference laboratory results in identifying resistance to rifampicin and isoniazid. Moreover, the BD MAX MDR-TB assay was comparable with the performance of the Xpert MTB/RIF assay [17]. In turn, in the study conducted by Shah et al., 1053 participants were recruited, and among patients with confirmed tuberculosis, the sensitivity of the BD MAX test was 93% (262/282), and the specificity was 97% (593/610) among participants with negative cultures on raw sputum samples. For samples that tested positive on fluorescence microscopy (smear-positive), the BD MAX test showed a sensitivity of 100% (175/175), whereas for smear-negative samples, the sensitivity was 81% (87/107). Sensitivity and specificity for rifampicin resistance obtained using BD MAX compared with phenotypic drug susceptibility testing were 90% and 95%, respectively. In turn, sensitivity for the detection of isoniazid resistance was lower (82%); however, the specificity was 100% [11]. Armstrong et al. assessed the diagnostic performance of the BD MAX assay for detecting MTBC and drug resistance in extrapulmonary samples spiked with MTBC from the Johns Hopkins strain collection. A total of 1083 tests were conducted on various sample types, showing an overall agreement rate of 94.8% (795/839) for detecting MTBC. For identifying resistance mutations, the agreement was 99% (379/383) for isoniazid and 96.4% (323/335) for rifampicin [18]. Another study published by Sagiroglu et al. [19] demonstrated a high correlation between *rpoB* mutations detected by BD MAX and phenotypic resistance to rifampicin. This aligns with our results, which also showed a 100% concordance between molecular and phenotypic rifampicin resistance detection. However, it is important to highlight that while BD MAX provides a rapid and accurate diagnostic tool, it has certain operational limitations that may affect its application in different healthcare settings. BD MAX requires specialized laboratory equipment, trained personnel, and regular maintenance, which could limit its deployment in low-resource environments.

Regarding isoniazid resistance, the study noted that while the BD MAX test efficiently detected the majority of resistant strains, cases leading to isoniazid resistance were missed. This resulted in a lower concordance rate (sensitivity = 71.4%) compared to rifampicin resistance detection. Similarly, our findings show a lower agreement between BD MAX and phenotypic AST results for isoniazid resistance, with discrepancies in four samples. Additional testing using Xpert MTB/XDR identified mutations in the *ahpC* and *fabG1* genes in these cases, highlighting the limitations of BD MAX in detecting certain isoniazid resistance mechanisms. The results of the study conducted by Ko et al. [20] are particularly interesting, as they yielded divergent results compared to ours and those of the other researchers mentioned above. Korean scientists showed that sensitivity for detecting isoniazid resistance was 100% using BD MAX. This contrasts with our findings, where four phenotypically resistant strains were not identified as resistant by BD MAX, as mentioned earlier. Interestingly, the same study reported that BD MAX had a sensitivity of only 50% in detecting rifampicin resistance, with one false-negative result, which differs from our results, where a 100% correlation was observed between *rpoB* mutations detected by BD MAX and phenotypic resistance to rifampicin. These discrepancies highlight the importance of the local validation of molecular assays before their widespread implementation. In addition, evaluating the cost-effectiveness of introducing BD MAX into national tuberculosis control programs is crucial. Although BD MAX requires significant initial investment for equipment and operational costs, its rapid turnaround time, providing results within 4 h, can lead to faster treatment initiation, which may reduce transmission rates and improve patient outcomes. For low- and middle-income settings, combining BD MAX with more affordable phenotypic tests can optimize resource management, enhancing diagnostic accuracy while maintaining cost-effectiveness. By improving early detection and treatment outcomes, BD MAX has the potential to reduce the burden of multidrug-resistant tuberculosis. Moreover, integrating rapid molecular tests into existing TB control programs can significantly enhance disease management efficiency and contribute to long-term savings in healthcare by preventing the spread of resistant strains. Sample type, sample amount, or mutation prevalence may also influence the observed differences. The factors leading to false-negative results in molecular methods for detecting rifampicin resistance have been infrequently examined. However, previous research has pointed out that some strains with the L533P mutation remain susceptible by molecular test. Furthermore, around 4% of rifampicin resistance cases are attributed to mutations located outside the *rpoB* gene [21,22,23]. These findings indicate that while BD MAX performs well in detecting drug-resistant TB, its accuracy can vary based on mutation distribution.

To summarize, the study results confirm the high effectiveness of the BD MAX MDR-TB test in detecting rifampicin resistance while also highlighting its limitations in identifying the full spectrum of mutations associated with isoniazid resistance. It was demonstrated that mutations in *ahpC* and *fabG1*, which are not detected by BD MAX MDR-TB, can lead to phenotypic isoniazid resistance, emphasizing the need for additional molecular tests in the diagnosis of drug-resistant tuberculosis.

## 5. Conclusions

The BD MAX MDR-TB molecular test demonstrates significant potential for the rapid and accurate diagnosis of tuberculosis, including the detection of multidrug-resistant strains, within 4 h of sample collection. Thanks to the short waiting time for results, clinicians can promptly initiate targeted treatment, particularly for patients with multidrug-resistant tuberculosis. However, to enable comprehensive clinical decision-making, it is essential to confirm the results of molecular resistance detection using traditional drug susceptibility testing methods. While BD MAX provides valuable insights into drug resistance, it has limitations in detecting the full spectrum of mutations, particularly those related to isoniazid resistance. Therefore, incorporating additional diagnostic tools, such as Xpert MTB/XDR or WGS, into standard diagnostic protocols could enhance resistance profiling and improve treatment outcomes

Future research should focus on improving the BD MAX test to detect a broader range of resistance mutations, as well as examining its application in diverse populations and settings. In addition, the cost-effectiveness and feasibility of integrating the BD MAX assay with other molecular or phenotypic tests in resource-limited settings should be carefully evaluated. This approach would enable more effective tuberculosis control, reduce the transmission of drug-resistant strains, and contribute to global health strategies aimed at combating multidrug-resistant tuberculosis.

## Figures and Tables

**Table 1:** Status of the investigated samples revealed by BD MAX MDR-TB test (*n* = 638).

BD MAX MDR-TB Status	*n*	%
Negative	550	86.2
Positive	79	12.4
Unresolved	9	1.4
Total number of samples	638	100.0

**Table 2:** Status of mutations found among the investigated positive samples (*n* = 79).

BD MAX—Mutations Status		*n*	%
None	Mutation/s type	42	53.2
Detected	*rpoB*	15	19.0
	*inhA/katG*	21	26.6
	*rpoB* and *inhA/katG*	14	17.7
	*rpoB* or *inhA/katG*	22	27.8
Unresolved		15	19.0

**Table 3:** Correlation between mutations status and successful AST results (*n* = 65).

BD MAX MDR-TB Mutations Status	AST Result		
	RIF-R/INH-R	RIF-S/INH-R	RIF-S/INH-S
*rpoB*(+) and *inhA/katG*(+)	14	0	0
*rpoB*(+) and *inhA/katG*(−)	1	0	0
*rpoB*(−) and *inhA/katG*(+)	0	4 (LRL)	0
		3 (HRL)	
*rpoB*(−) and *inhA/katG*(−)	0	2 (LRL)	40
		1 (HRL)	
Total	15	10	40

AST—antimicrobial susceptibility testing, HRL—high resistance level, INH—isoniazid, LRL—low resistance level, R—resistant, RIF—rifampicin, S—sensitive.

**Table 4:** Correlation between *rpoB* gene mutations and AST for rifampicin.

	*rpoB* Gene Mutations	AST Status for Rifampicin			
		*n*	S	R	Unknown *
BD MAX status	positive	15	0	15	0
	negative	50	47	0	3
	unreported	14	10	0	4

*—no growth in a culture resulting in AST results lack, AST—antimicrobial susceptibility testing, R—resistant, S—sensitive.

**Table 5:** Correlation between *inhA/katG* genes mutations and AST for isoniazid.

	*inhA/katG* Genes Mutations		AST Status for Isoniazid		
		*n*	S	R	Unknown *
BD MAX status	positive	21	0	21	0
	negative	44	37	4	3
	unreported	14	9	0	5

*—no growth in a culture resulting in AST results lack, AST—antimicrobial susceptibility testing, R—resistant, S—sensitive.

## Data Availability

The data presented in this study are available upon request from the corresponding author.

## Supplementary Materials

The following supporting information can be downloaded at: https://www.mdpi.com/article/10.3390/pathogens14060602/s1, Table S1: The detailed origin of the samples included into the study (*n* = 638).

## Abbreviations

The following abbreviations are used in this manuscript:

IGRA	interferon gamma release assay
INH	isoniazid
KatG	catalase-peroxidase
MDR	multidrug-resistant
MDR-TB	multidrug-resistant tuberculosis
MTBC	*Mycobacterium tuberculosis* complex
NAD	nicotinamide adenine dinucleotide
NAAT	nucleic acid amplification tests
NGS	next-generation sequencing
TST	tuberculin skin tests
qPCR	real-time PCR
RIF	rifampicin
TB	tuberculosis
WGS	whole-genome sequencing
