# Supplementary material for: The Usefulness of the BD MAX MDR-TB Molecular Test in the Rapid Diagnosis of Multidrug-Resistant Tuberculosis
Source: Pathogens. 2025 Jun 19;14(6):602. doi: 10.3390/pathogens14060602 (PMC12195635; doi:10.3390/pathogens14060602)
Supplement: Supplementary file 1 [file pathogens-14-00602-s001.zip › pathogens-3573875-supplementary.pdf]

Supplementary Materials

Table S1. The detailed origin of the samples included into the study (*n* = 638)

Specimen type	n	%
Broncho-alveolar lavage	313	49.1
Sputum	155	24.3
Pleural fluid	84	13.2
Fragments of tissues (31), bones (2), nodes aspirate (1)	34	5.3
Urine	19	3.0
Other body fluids (peritoneal fluid - 5, pericardial fluid - 5, fluid from the cyst - 2, cerebrospinal fluid - 1, fluid from the abdominal cavity - 1, bile - 1)	15	2.4
Gastric lavage	14	2.2
Pus (2) and swabs (wound - 1, ear - 1)	4	0.6
Total	638	100.0
